# Four Atypical Parathyroid Adenomas Detected by Dual Phase Tc-99m MIBI SPECT

**DOI:** 10.4274/mirt.galenos.2019.09326

**Published:** 2020-02-17

**Authors:** Mine Araz, Derya Çayır, Fatma Fulya Köybaşıoğlu, Harun Karabacak, Erman Çakal

**Affiliations:** 1 University of Health Sciences, Dışkapı Yıldırım Beyazıt Training and Research Hospital, Clinic of Nuclear Medicine, Ankara, Turkey; 2 University of Health Sciences, Dışkapı Yıldırım Beyazıt Training and Research Hospital, Clinic of Pathology, Ankara, Turkey; 3 University of Health Sciences, Dışkapı Yıldırım Beyazıt Training and Research Hospital, Clinic of General Surgery, Ankara, Turkey; 4 University of Health Sciences, Dışkapı Yıldırım Beyazıt Training and Research Hospital, Clinic of Endocrinology and Metabolism Diseases, Ankara, Turkey

**Keywords:** Parathyroid gland, parathyroid adenoma, Tc-99m sestamibi, single photon emission computed tomography

## Abstract

We report a case of a 55-year-old female with tertiary hyperparathyroidism and osteoporosis who had end stage renal disease and a history of hemodialysis for 15 years. Patient’s informed is taken. Neck ultrasonography showed multinodular goiter together with a hypoechoic lesion compatible with a parathyroid adenoma. Dual phase technetium (Tc) Tc-99m MIBI single photon emission computed tomography (SPECT) showed pathological uptake in four parathyroid gland locations. Total thyroidectomy and subtotal parathyroidectomy revealed nodular hyperplasia and atypical adenomas in four parathyroid glands. Atypical parathyroid adenoma is a rare clinical entity. Multiple atypical adenomas are even less frequent. A very rare condition, detection of atypical adenomas in four of the parathyroid glands by dual phase Tc-99m MIBI SPECT, is presented in this case.

## Abstract (tr)

Son dönem böbrek hastalığı nedeni ile 15 yıldır hemodiyalize giren, tersiyer hiperparatiroidi ve osteoporozu bulunan 55 yaşında bir kadın hastayı sunuyoruz. Hastadan bilgilendirilmiş onam alınmıştır. Hastanın boyun ultrasonografisinde multinodüler guatr ve paratiroid adenomu ile uyumlu olan hipoekoik bir lezyon saptandı. Dual faz teknesyum (Tc) Tc-99m MIBI tek foton emisyonlu bilgisayarlı tomografi (SPECT), dört paratiroid bezi lokalizasyonunda patolojik tutulum gösterdi. Total tiroidektomi ve subtotal paratiroidektomi sonucu tiroid bezinde nodüler hiperplazi ve dört paratiroid bezinde atipik paratiroid adenomu ile uyumluydu. Atipik paratiroid adenomu nadir görülen bir klinik antitedir. Multipl atipik paratiroid adenomu daha da az sıklıkla görülür. Bu olguda, dört atipik paratiroid adenomunun dual faz Tc-99m MIBI SPECT ile saptandığı çok nadir bir durumu sunuyoruz.

## Figures and Tables

**Figure 1:**
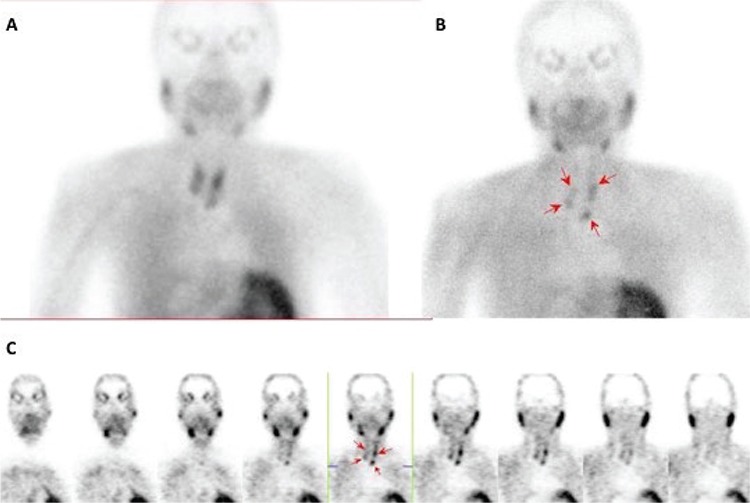
Dual phase technetium (Tc) Tc-99m MIBI planar imaging and single photon emission computed tomography (SPECT) (Figure 1A) were performed in nuclear medicine department. Following intravenous injection of 20 mCi Tc-99m MIBI, early (15 min after injection) (Figure 1B) and delayed (2 hours later) (Figure 1C) planar images of the neck and upper abdomen were obtained by a large field of view γ cammera equipped with a low energy high resolution parallel hole collimator. SPECT study was performed 2 hours after radiopharmaceutical injection (images were acquired at 120 projections, 20 sec/projection, into 128x128 matrix) revealed focal activity retention in four different locations: Posterior neighbourhood of the middle portion and lower pole of the left lobe and posterior neighbourhood of the middle portion and lower pole of the right lobe. Scintigraphic findings were interpreted as supportive of parathyroid hyperplasia secondary to end stage renal disease.

**Figure 2:**
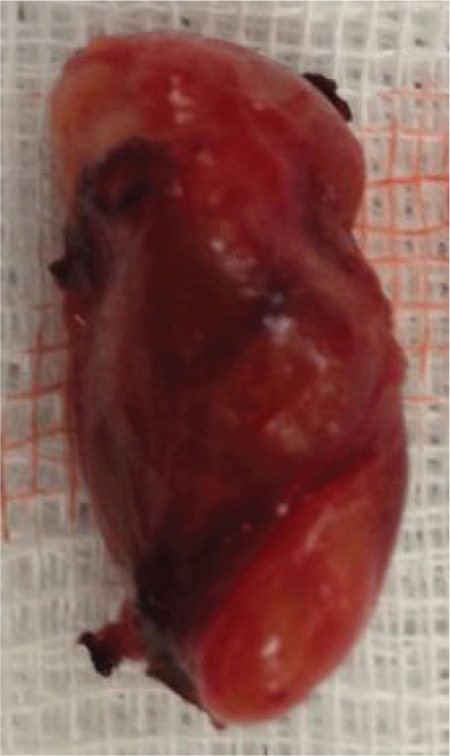
Surgery involving total thyroidectomy and removal of 3 and a half of the parathyroid glands was performed. Macroscopic demonstration of the left inferior parathyroid gland is given.

**Figure 3:**
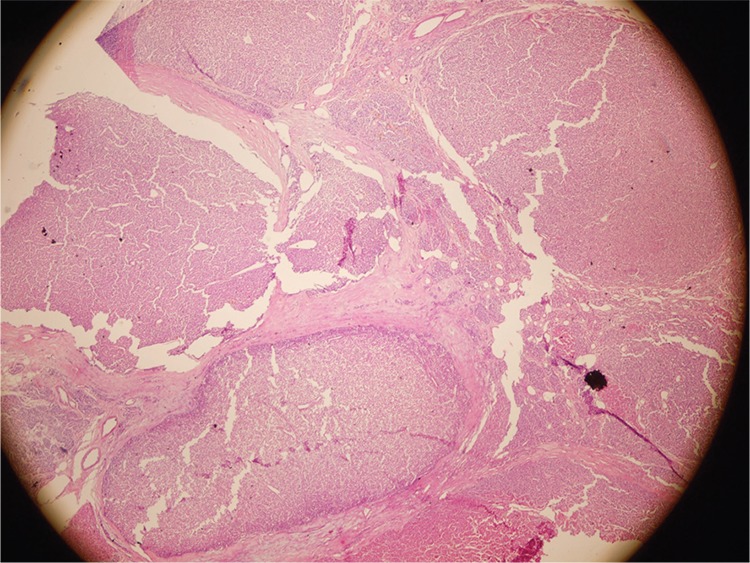
Histopathological examination revealed nodular hyperplasia of thyroid gland and atypical parathyroid adenomas of four glands. Nodular structures were seperated by fibrous septae. No typical findings suggestive of parathyroid carcinoma like mitosis, atypia or vascular invasion were observed (hematoxylin and eosin, magnification x400). Preoperatively, serum parathormone (PTH) was 1876 pg/mL, Ca was 10.14 mg/dL and P was 5.02 mg/dL. Following the operation, serum PTH level dropped to 99 pg/mL and serum Ca level was 9.7 mg/dL. Tertiary hyperparathyroidism is mostly caused by hyperplasia and rarely by adenoma (1). Atypical parathyroid adenoma is a rare entity with borderline pathological characteristics, between adenoma and carcinoma (2). Parathyroid surgery is a relatively hard procedure as it necessitates surgical skill and experience. The use of Tc-99m MIBI scintigraphy is well established for patients undergoing reoperation for hyperparathyroidism. Some surgeons tend to skip preoperative imaging in secondary or tertiary hyperparathyroidism because bilateral neck exploration is needed anyway, but some studies suggested that parathyroid scintigraphy could be of value before initial parathyroidectomy (3,4). Tc-99m MIBI scintigraphy may help recognize an unexpected appearance of an ectopic or supernumerary parathyroid gland, as well as show the gland with the least radiotracer accumulation that can be autotransplanted. Protocol of parathyroid scan is argued to be important. Although dual isotope techniques are reported to be superior to dual phase imaging and SPECT/CT is recommended strongly to provide topographic anatomic information, in this case, both four pathological glands could still be identified by dual phase Tc-99m MIBI SPECT (5). Ultrasonography (USG) and Tc-99m MIBI scintigraphy are complementary in parathyroid imaging. The success of both modalities is similar in single gland disease. However, in case of multiglandular disease, frequently seen in secondary or tertiary hyperparathyroidism, they have been reported to have lower sensitivities. Thus, especially in these patients, combination of scintigraphic and sonographic imaging provides a more accurate approach in the preoperative evaluation (6). In our case, MIBI was capable of detecting atypical adenomas in four parathyroid glands with respect to one gland demonstrated by USG. This report is interesting in the way that atypical adenomas of parathyroid gland were presented in all four of the glands in a patient with tertiary hyperparathyroidism and that the only preoperative method that could address multiglandular disease was Tc-99m MIBI SPECT.

## References

[1] Sheu-Grabellus SY, Schmid KW (2015). Pathology of parathyroid glands: Practical aspects for routine pathological investigations. Pathologe.

[2] Carlson D (2010). Parathyroid pathology: hyperparathyroidism and parathyroid tumors. Arch Pathol Lab Med.

[3] Piga M, Bolasco P, Satta L, Altieri P, Loi G, Nicolosi A, Tarquini A, Mariotti S (1996). Double phase phase parathyroid technetium-99m- MIBI scintigraphy to identify functional anatomy in secondary hyperparathroidism. J Nucl Med.

[4] Torregrosa JV, Fernández-Cruz L, Canalejo A, Vidal S, Astudillo E, Almaden Y, Pons F, Rodriguez M (2000). (99m)Tc-sestamibi scintigraphy and cell cycle in parathyroid glands of secondary hyperparathyroidism. World J Surg.

[5] Taïeb D, Ureña-Torres P, Zanotti-Fregonara P, Rubello D, Ferretti A, Henter I, Henry JF, Schiavi F, Opocher G, Blickman JG, Colletti PM, Hindié E (2013). Parathyroid scintigraphy in renal hyperparathyroidism: the added diagnostic value of SPECT and SPECT/CT. Clin Nucl Med.

[6] Ruda JM, Stack BC Jr, Hollenbeak CS (2006). The cost-effectiveness of additional preoperative ultrasonography or sestamibi-SPECT in patients with primary hyperparathyroidism and negative findings on sestamibi scans. Arch Otolaryngol Head Neck Surg.

